# A New Laparoscopic Manoeuvre in Median Arcuate Ligament Syndrome

**DOI:** 10.4274/balkanmedj.2017.0596

**Published:** 2017-12-01

**Authors:** Selçuk Gülmez, Ulaş Aday, Aziz Serkan Senger, Ebubekir Gündeş

**Affiliations:** 1 Department of Gastroenterological Surgery, University of Health Sciences, Kartal Koşuyolu High Specialized Training and Research Hospital, İstanbul, Turkey

## To The Editor,

Median Arcuate Ligament syndrome is a rare cause of chronic gastrointestinal ischemia (1). Anatomically, median arcuate ligament is a musculofibrous structure uniting both diaphragmatic crura from the front at the aortic hiatus level (2). Abnormally downward located median arcuate ligament lies at the pathophysiology of this disease, and intestinal angina symptoms characterized by postprandial pain, nausea-vomiting and weight loss manifest themselves due to chronic compression the celiac artery (3).

Median Arcuate Ligament syndrome requires surgical treatment in symptomatic patients (4). The conversion rate of Median Arcuate Ligament syndrome remains between 13% and 27%, and the main reason for conversion is haemorrhaging related to vascular damage (5). Standardization of the technique will contribute to decreasing these rates, but no standard surgical technique has yet been set. The most critical stage of this procedure proves to be the dissection of truncus coeliacus. Therefore, our aim in this case report was to share our technique enabling the safe dissection of truncus coeliacus.

**Figure 1:**
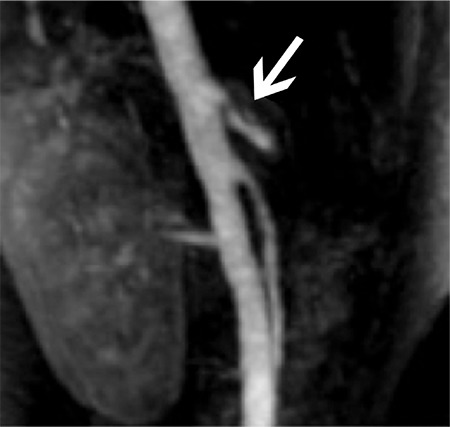
Preoperative magnetic resonance-angiography, stenosis of 2 mm and post-stenotic dilatation in truncus coeliacus.

A 20-year-old female patient presented to our clinic with complaints of classic Median Arcuate Ligament syndrome symptoms. The patient’s physical examination, preoperative laboratory results, gastroscopy and abdominal ultrasonography were normal. Her abdominal computed tomography showed suspected truncus coeliacus compression, magnetic resonance angiography was performed in order to confirm the diagnosis, and the findings were concordant with median arcuate ligament-related arterial stenosis of 2 mm and post-stenotic dilatation (Figure 1).

The patient was informed about the procedure and written consent was obtained. Median arcuate ligament was seperated by decompression laparoscopically and was started on oral intake on the first postoperative day, and all her existing complaints were eliminated. The patient was discharged from our clinic on the fourth postoperative day. She had no complaints in the follow-up control done 6 months after discharge.

**Figure 2:**
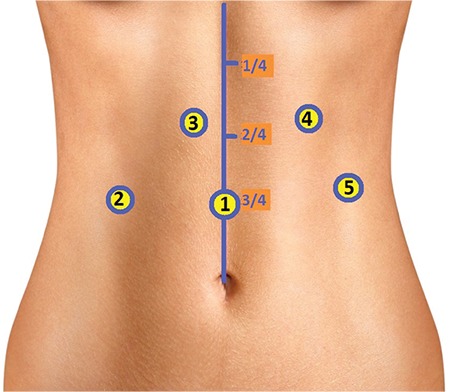
Port sites. Port 1: 10 mm, camera, ¾ caudal of the distance between the belly and the xiphoid; Port 2: 5 mm, liver retraction; Ports 3 and 4: 5 mm, working; Port 5: 5 mm, esophageal traction

**Figure 3:**
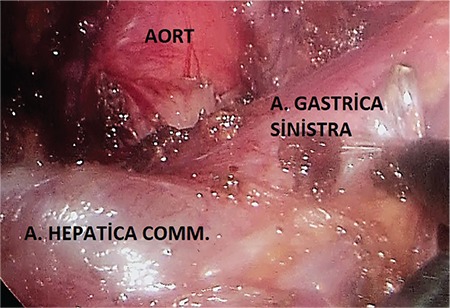
The initial imaging of the truncus coeliacus before the maneuver.

**Figure 4:**
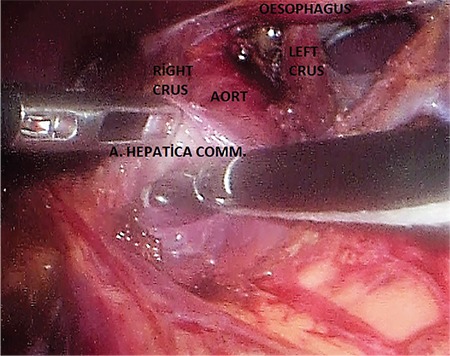
The truncus coeliacus following the traction of hepatic and left gastric arteries by type.

**Figure 5. a-c:**
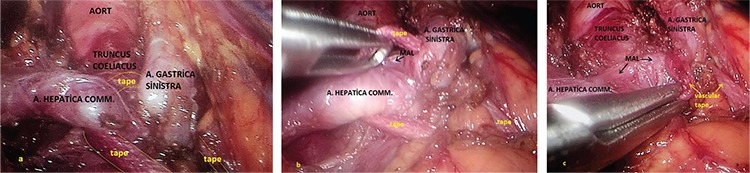
The final maneuver, Visuality problem of the truncus coeliacus when the tape was downwards tractioned (a), The traction of the vascular tape from the midpoint downwards and the isolation of median arcuate ligament (b, c).

**Laparoscopic technique:** Ports were placed and the patient was laid in the 30° reverse-Trendelenburg position (Figure 2). The right crus was found by opening up the gastrohepatic ligament. The oesophagus was suspended by penrose, tractioned towards the patient’s left and placed in the middle on the left crus to the posterior of the oesophagus. The fibrous fibres on the aorta were separated by hook cautery from proximal to distal part. When the truncus level has been reached, arteria hepatica communis and the left gastric artery, which became easily identified because of the prestenotic dilation, were prepared from the exit site, but the splenic artery was not isolated, as it was the branch of truncus coeliacus reaching to the caudal part of pancreas. The truncus coeliacus could not be clearly evaluated because it remained right angled despite the 30° of optics (Figure 3). An additional manoeuvre was necessary at this point in order to continue with the procedure safely. We transitioned to a retrograde approach at this point from the distal side upwards, and both vessels were suspended together with the vascular tape (Figure 4), but it was observed that the hepatic and left gastric artery made an angle rendering the isolation of the truncus coeliacus challenging when the tape was tractioned (Figure 5a). The surgeon, at this final stage of our maneuver, tractioned the tape by holding it at the midpoint of the intersection of the hepatic and left gastric arteries towards the distal side by means of the endograsper/endoclinch (Figure 5b, 5c). Then, the celiac plexus ganglion fibres and lymphatics alongside with the median arcuate ligament on the truncus coeliacus were cut by using hook cautery.

We believe that isolation of median arcuate ligament can be facilitated when it is seen clearly, and vascular damage- related complications can be decreased thanks to the maneuver, which offers a simple and efficient solution.
